# Patient Safety Incidents and the second victim phenomenon among nursing students

**DOI:** 10.1590/1980-220X-REEUSP-2022-0005en

**Published:** 2022-10-10

**Authors:** Ana Paula Mousinho Tavares, Jamila Geri Tomaschewski Barlem, Laurelize Pereira Rocha, Aline Cristina Calçada de Oliveira, Fernanda Valéria Silva Dantas Avelino, Gabriela do Rosário Paloski

**Affiliations:** 1 Universidade Federal de Pelotas, Faculdade de Enfermagem. Pelotas, RS, Brazil.; 2 Universidade Federal do Rio Grande, Escola de Enfermagem, Programa de Pós-graduação em Enfermagem. Rio Grande, RS, Brazil.; 3 Universidade Federal do Piauí, Programa de Pós-Graduação em enfermagem. Teresina, Piauí, Brazil.

**Keywords:** Students, nursing, Education, nursing, Patient Safety, Safety management, Clinical Clerkship, Estudiantes de Enfermería, Educación en Enfermería, Seguridad del Paciente, Administración de la Seguridad, Prácticas clínicas, Estudantes de enfermagem, Educação em enfermagem, Segurança do paciente, Gestão de segurança, Estágio clínico

## Abstract

**Objective:**

To map the factors involved in incidents that harm patient safety and contribute to the second victim phenomenon among nursing students.

**Method:**

Qualitative, exploratory-descriptive study addressing 23 nursing students attending a Federal University in the South of Brazil. The interviews were analyzed using text and discoursive analysis. The Iramuteq software supported the processing of texts.

**Results:**

Communication failures within the health staff, a lack of protocols and equipment that prioritize patient safety, and factors related to the teaching-learning process favor the occurrence of patient safety incidents and the second victim phenomenon among nursing students.

**Conclusion:**

Addressing the topic concerning patient safety in nursing programs can promote the patient safety culture by encouraging reporting and admitting the possibility of errors and learning from them, strategies that can mitigate second victim effects.

## RESUMO

**Objetivo:**

mapear os fatores envolvidos em incidentes que fragilizam a segurança dos pacientes e que colaboram para o fenômeno da segunda vítima entre os estudantes de enfermagem.

**Método:**

estudo de abordagem qualitativa, de caráter exploratório-descritivo, realizado com 23 estudantes de enfermagem de uma Universidade Federal do Sul do Brasil. As entrevistas foram analisadas através da análise textual discursiva. O software Iramuteq auxiliou no processamento dos textos.

**Resultado:**

falhas na comunicação entre a equipe de saúde, ausência de protocolos e de equipamentos que priorizem a segurança do paciente e os fatores relacionados ao processo de ensino-aprendizagem favorecem a ocorrência de incidentes de segurança do paciente e corroboram para o fenômeno da segunda vítima entre estudantes de enfermagem.

**Conclusão:**

a incorporação da temática sobre segurança do paciente no curso de enfermagem pode auxiliar o desenvolvimento de uma cultura de segurança do paciente, ao incentivar a cultura do relato, admitir a possibilidade do erro e o aprendizado a partir dele, estratégias que podem propiciar na mitigação dos efeitos da segunda vítima.

## RESUMEN

**Objetivo:**

mapear los factores que participan en incidentes que fragilizan la seguridad de los pacientes y que colaboran para el fenómeno de la segunda víctima entre los estudiantes de enfermería.

**Método:**

estudio de abordaje cualitativo, de carácter exploratorio y descriptivo, realizado en 23 estudiantes de enfermería de una Universidad Federal del sur de Brasil. Las entrevistas fueron analizadas a través del análisis textual discursivo. El *software* Iramuteq auxilió en el procesamiento de los textos.

**Resultado:**

las fallas en la comunicación entre el equipo de la salud; la ausencia de protocolos y de equipamientos que den prioridad a la seguridad del paciente; y, los factores relacionados al proceso de enseñanza y aprendizaje, favorecen la ocurrencia de incidentes de seguridad del paciente y corroboran el fenómeno de la segunda víctima entre estudiantes de enfermería.

**Conclusión:**

la incorporación de la temática sobre seguridad del paciente, en el curso de enfermería, puede auxiliar a desarrollar una cultura de seguridad del paciente al incentivar la cultura del relato, admitir la posibilidad del error e incentivar el aprendizaje; y, partiendo de este, crear estrategias que puedan auxiliar a mitigar los efectos de la segunda víctima.

## INTRODUCTION

Entering a university represents a time of intense changes in students’ lives. There is great expectation of new experiences, learning, and relationships, but these expectations are also accompanied by fear and insecurity toward this new context. Most students are young and just left high school, and somewhat immature and face the dilemma of choosing a profession in addition to the responsibility of starting a higher education program^(1)^.

The academic path within the program, experiences, relationships, and bonds established during the undergraduate program, as well as the methodologies that the professors adopt, determine whether these individuals will remain in the profession and the direction they will follow after graduation. A professor, or even a class, may motivate and inspire a student to become a specialist in a specific field within healthcare, encourage him/her to pursue teaching or research, or be responsible for a student interrupting or quitting the program. All these outcomes, coupled with personal needs, are influenced by the experiences and relationships within academia^(2)^.

Nursing is a predominantly practical program in which students begin their training with theoretical axes and gradually enter healthcare services with practical classes, ending with supervised internships. Training in a clinical setting involves the participation of students, professors, and clinical nurses. Hence, students are supervised and receive structured training in the clinical environment and have the opportunity to acquire and develop communication, anamnesis, physical examination, clinical reasoning, diagnosis, and management skills^(3)^.

Therefore, the practical field allows students to associate theoretical knowledge with practice, learn new procedures, improve attitudes and interpersonal relationship skills, and recognize their professional identity. However, practical classes and internships are experiences that generate great expectations and anxiety among students because it is when they have their first contact with patients. Hence, students’ distress results from insecurity and fear of making mistakes, which are prevalent feelings^(4)^.

Nursing students have the potential, even if to a lesser extent, to become second victims when they are introduced to the clinical and hospital setting. Therefore, the internship elicits mixed feelings because they have the opportunity to put their knowledge and skills into practice, but it is also a time of great anxiety, as they fear causing harm to a patient in case they are involved in a patient safety incident^(4)^.

The pressure imposed during training by professors, colleagues, healthcare staff, and even society may magnify the fear of making mistakes. The perception that good professionals do not err and those who do are not good still prevails in the health field, reinforcing a punitive culture of safety, with an emphasis on individual blaming whenever an incident occurs^(5)^.

Patient safety culture must be strengthened in the training process of undergraduate nursing students. It can be defined as the result of individual and group values, attitudes, perceptions, competencies, and behavior patterns, which holds everyone, professionals and patients, accountable for their safety and the safety of patients, prioritizes safe and quality care above all other goals, and encourages the identification, reporting, and resolution of patient safety issues^(6,7)^.

Strengthening patient safety culture among students may help mitigate the adverse effects related to patient safety incidents, i.e., events or circumstances that cause, or may cause, unnecessary harm to a patient^(5)^. When those responsible for care are involved in these events, physical and/or psychological symptoms may be associated. In this context, the term “second victim” emerged, which refers to health professionals who experience distress or trauma after being directly involved with an incident^(8)^.

Later, the term “second victim” was expanded to include healthcare providers directly or indirectly involved in an adverse event or unexpected action, experiencing trauma and distress. Because this definition is broader, it was adopted as a reference for this study; thus, professionals from different fields, students, and professors, can be considered second victims^(9)^.

From this perspective, the term “third victim” refers to health organizations, management professionals, and educational institutions that may suffer adverse effects even though they are not directly involved with^(10)^.

The second victim’s symptoms, such as post-traumatic stress, and emotional and psychological exhaustion, can be even more exacerbated in nursing students still undergoing professional training. In addition, being involved in a patient safety incident may lead to dissatisfaction with the profession, raise questions about whether the profession was the right choice, cause mood disorders or medication use, and even lead students to quit the program^(11)^.

In this sense, this study’s research question is: what are the factors involved in incidents that weaken patient safety and contribute to the second victim phenomenon among nursing students? Hence, this study aimed to map the factors involved in incidents that weaken patient safety and contribute to the second victim phenomenon among nursing students.

## METHOD

### Study Design

This is a qualitative study of an exploratory-descriptive nature. The Consolidated criteria for reporting qualitative research (COREQ) were followed in this study according to the guidelines established for studies with a qualitative approach.

### Population

The sample comprised 23 nursing students attending the university’s ninth or tenth semester of the Undergraduate Nursing Program. The number of students regularly enrolled during data collection, from March to April 2021, was 15 in the ninth and 21 in the tenth semester. After an invitation to participate in the study, the sample included 23 students: nine students attending the ninth semester and 11 students in the tenth semester; the researcher considered that the data collected met the objectives and reached saturation.

### Study Setting

The study was performed in the Undergraduate Nursing program of a Federal University located in the South of Rio Grande do Sul, Brazil. The settings included the medical clinic, surgical clinic, traumatology, and maternity units at the university hospital linked to the University, where the students were attending the supervised clinical internship under the technical supervision of nurses from their respective sectors, units or services, and under the guidance of nursing professors from the School of Nursing.

The supervised internship of the nursing program refers to professional practice activities implemented in the program’s last two semesters (ninth and tenth semesters). The ninth semester’s supervised internship comprises 420 hours and corresponds to four essential hospital practice courses: emphasis on women’s health, emphasis on child and adolescent health, emphasis on adult and elderly health, and emphasis on perioperative nursing. In turn, the tenth-semester internship comprises 540 hours and is structured into two courses: Primary Health Care and a field the students can choose.

### Selection Criteria

The inclusion criteria adopted to select the participants were: being an undergraduate student attending the nursing program and regularly enrolled in the ninth or tenth semester, working in health facilities. The exclusion criteria were being absent due to temporarily interrupting the program or academic mobility during data collection.

### Sampling

Non-probabilistic convenience sampling was used to select the participants; that is, the participants were not randomly assigned but chosen according to the characteristics of the study’s group. Hence, the participants were selected according to their presence and availability during data collection.

### Data Collection

Face-to-face and online semi-structured interviews were used to collect data. Eighteen people were interviewed in person in a private room on the university hospital’s premises. All sanitary and distancing measures and protocols were adopted, including facemasks and alcohol. In addition, five students agreed to participate in the study through a previously scheduled online interview via video call.

The students were approached during the internship last week, and the interviews were recorded using a digital electronic device after the participants authorized and signed free, informed consent forms. The researcher transcribed the 20-minute interviews. Data were collected between March and April 2021.

Additionally, a questionnaire that had been previously developed was validated to verify whether the participants would understand the questions and analyze whether the questions would be sufficient to meet this study’s objectives. Thus, before starting data collection, the questionnaire was tested with two participants excluded from the final sample.

The questionnaire comprised close-ended questions to characterize the participants and open-ended questions addressing aspects related to patient safety incidents and the second victim phenomenon. The questionnaire initially addresses the students’ general perceptions of incidents and errors in the healthcare context. This question was chosen to initiate the interview because we believe it would make the students more comfortable, considering it is a delicate subject that may cause discomfort and elicit unwanted memories.

### Data Analysis and Treatment

The interviews were submitted to text and discourse analysis, described according to three stages: unitarization, categorization, and capture of a newly emerging understanding. First, the text was deconstructed and separated into units of meaning and then grouped into categories to support the construction of a metatext containing understandings that emerged from the phenomenon under study^(12)^.

The IRAMUTEQ software, which presents several forms of analysis, was used to process data. IRAMUTEQ’s most complete form of analysis was used in this study. It consists of the **REINERT method** or **Descending Hierarchical Class (CHD)**. First, the texts, called *corpus*, were classified according to their respective vocabularies. This set of texts was then divided according to the frequency in which reduced forms appeared. Finally, the CHD method was applied considering matrices that crossed text segments (TS) and words (repeated X^2^ test with p < 0.05) to obtain a stable and definitive classification^(13)^.

The software analyzed and fractionated the *corpus* through statistical calculations until reaching TS. Hence, IRAMUTEQ facilitates the first phase of the analysis by separating the corpus into TS and uniting them into units of meaning. The classes the software created are composed of these TS, based on their classification and vocabulary distribution.

**Figure 1.**
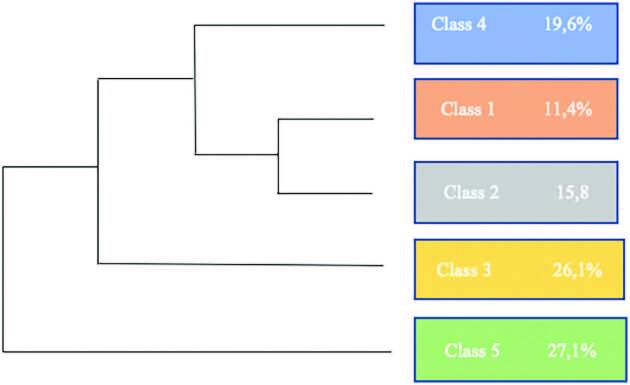
Dendrogram of the Descending Hierarchical Classification. Rio Grande, RS, Brazil, 2021.

IRAMUTEQ recognized the separation of the *corpus* consisting of 23 texts into 937 TS, in which 839 TS (89.54%) remained. The dendrogram below emerged from the classes IRAMUTEQ identified and their relationships with each other (Figure 1).

The following categories emerged: “Nursing students as the second victim of unsafe healthcare systems” (class 5) and the “Influence of the teaching-learning process on the second victim phenomenon” (classes 1 and 2).

### Ethical Aspects

All the precepts established in Resolution No. 510 from April 7, 2016, of the Brazilian National Health Council concerning ethical aspects regulating research in Human and Social Sciences were complied with, together with the official letter that regulates the collection of data on online, Letter No. 2/2021/CONEP/SECNS/MS. The Institutional Review Board at the Federal University approved this study (Opinion report 4,487,347) in 2020. Letter “S” followed by a sequential number, “S1, S2, S3…” was used to ensure the participants’ confidentiality. The interviews were resumed with each participant to ratify the transcriptions’ authenticity. All voluntary participants signed free and informed consent forms or had the form sent by e-mail.

## RESULTS

Twenty-three nursing students participated in the study: nine attended the ninth semester, and 14 attended the tenth semester. All participants were female and aged between 20 and 46. Only four students reported training and experience as nursing technicians.

## NURSING STUDENTS AS THE SECOND VICTIM OF UNSAFE HEALTHCARE SYSTEMS

This category revealed the incidents the nursing students experienced during hospital care and their relation with the second victim phenomenon. The reported events might be related to an unsafe healthcare system, which lacks barriers to prevent errors that contribute to patient safety incidents, putting students at risk of becoming second victims.

Communication failures within the health staff and a lack of protocols and equipment that prioritize patient safety were found to be the main factors related to the involvement of students in patient safety incidents and, consequently, triggered the second victim phenomenon.

*Sometimes the hospital misleads us, so sometimes you move between units that do not work in the same way (…) we are afraid, but we don’t know the material, don’t know the color system; even if they are labeled, we are not used to that, so sometimes the material the hospital is providing is the same equipment that can be connected to two very different inputs, so, I think this is also a factor* (S1).

*I believe that it is not just the professional who plays a role, but sometimes it’s a device that is confusing, or similar medications, (…) also, the professionals who sometimes are tired* (S2).

*The practices, in this case, differ from one sector to another; there are no standards, which can also lead to errors and cause patients pain* (S21).

(…) *they had already given the patient the medication, and I gave it again. Nothing happened, but there was a problem there, of not checking, of them not having passed the shift* (S2).

*I told her that we only needed to make an identification label, that I had just aspired diluted adrenaline and pure adrenaline, which the pediatrician had asked for. She went there and took my two adrenaline pumps. The technician with me saw that I had aspirated it, but the nurse threw both adrenaline out, and we had no more adrenaline in the sector. The baby was born with no pulse, they took him away, and the technician had to ask the anesthesiologist to give him adrenaline* (S2).

*It has already happened to me, for example, delaying medication. So, I sort of ended up getting involved with things and left it for later. I’ve also had a patient with the same name in the same room; only their last names were different…* (S7).

Additionally, among the factors influencing the involvement of students in incidents, lack of teamwork, work overload, and personal factors stand out. Also, not trusting nursing students and the presence of conflicts within the team were reported as predictors that students would experience risk situations:

*He didn’t even believe the technician who said that I had just aspired adrenaline. The technician told me that the nurse, who was new to the sector, didn’t have confidence in the team, and that’s why she ended up doing it* (S2).

*There were many people to attend to, there was only one professor, and the unit nurse was changing shifts, so I went by myself* (S21).

## INFLUENCE OF THE TEACHING-LEARNING PROCESS ON THE SECOND VICTIM PHENOMENON

This category comprises the factors related to the teaching-learning process and its contribution to the occurrence of the second victim phenomenon, with an emphasis on the punitive culture that still permeates the training environment, on the weaknesses of teaching patient safety, on the gap between theory and practice, and the role of professors and the communication process established with students.

The results show that the premise that errors cannot be part of health care is still supported in the teaching-learning process, hindering the implementation of the positive safety culture in the nurses’ training process. As a result, conceptions of individual guilt, punishment, and omission of errors are reinforced, favoring the second victim phenomenon.

*Even within the university itself, we are under pressure because we can never make mistakes, we always have to get it right, and this pressure begins from the moment we start the internship* (S12).

*Nobody was going to judge me, but I judged myself because I knew I was doing it wrong and I’ve also seen some students afraid of making mistakes and that the professor will judge them* (S23).

Lack of knowledge about basic aspects concerning patient safety and the management of incidents may also favor the emergence of the second victim phenomenon among students. The students feel unprepared to manage risks and incidents:

*If something happens, imagine what we will feel, you know, because, in our academic life, we are always very insecure* (S9).

*If you ask me, what I’m most afraid of right now is making mistakes because our mistakes in the health field can be fatal. It may be something silly, but I can’t tell you how I’d feel if I made some mistake*. (S4).

*We think, like, oh, I’m just going to get it right, just get it right, but suddenly something unexpected happens, something we hadn’t foreseen, and it totally destabilizes us, so don’t think about errors, but look out for a potential error* (S22).

Additionally, the students expressed insecurity and questioned the development of their professional skills, emphasizing the few opportunities they had to train their skills during the internship. Having many students and low demand for procedures influence the students’ perception of the gap between theory and practice. Additionally, some of the content is not addressed in the academy but is required in practice, which may result in future mistakes. Therefore, students also associate the possibility of incidents with what is taught in theory but which is often not translated into practice, leading to a feeling of guilt and intensifying personal and academic distress.

*We know that at some point, we will have to do it for the first time, only that we get here at the end of the program very insecure because I have never done some procedures. I’m at the end of the undergraduate program, and I don’t know basic things like how to handle an infusion pump, so I wonder if I’m in the right place, doing the right thing* (S19).

*We don’t learn this in college anymore. I think it’s necessary because when the technician doesn’t know it, they ask a nurse, so the nurse has to know it, and I think this may lead to an error eventually (…) get the saline solution and wash it because if you apply the gauze many times, the lesion will bleed, but then they said that the professor told them to apply the gauze many times. So I said, no, girls, in your practice, depending on the injury, it doesn’t work like that* (S23).

It is also worth noting that the professors’ attitude and their reactions toward a student involved in a patient safety incident may trigger and intensify personal and academic distress among students:

*I started to reason; I looked at the prescription and saw that the dose was correct, so since it was not something huge, I think she could have softened her reaction a bit because it upset us very much* (S19).

*I think it takes great skill to deal with students because we are here learning, even those who have been working for a long time are not exempt from making mistakes, and I think the professor should deal with this in private because it has repercussions. (…) I think that having a professor whom you know and trust, even when you are not well or when you are wrong, I think it is fundamental because if you create a barrier, after you create a barrier, then it’s over…* (S19).

Finally, communication barriers between professors and students are an important factor contributing to the second victim phenomenon. When students become involved in an incident and do not find supporting professors, they may suffer in silence and blame themselves for the event.

*People are afraid to come and say, look, I did it wrong; I don’t know how to do it. So people, especially at the beginning of college, are under much pressure* (S23).

*When I got home, I kept thinking that I was using saline solution and then dipyrone and that there was no fluid from the patient; then I calmed down, but we would not tell the professor because we were afraid* (S23).

## DISCUSSION

Among the safety incidents the nursing students reported, errors involving medication administration and nasoenteral diet stood out. This finding is similar to the one reported by a study conducted in a Nursing School in Belgium, in which the most frequent safety incidents were related to medication administration (34.2%), followed by falls (33.2%) and (19.4%), resulting from staff behavior and lack of attention^(11)^.

The students’ descriptions of how the incidents happened show factors besides the human factor that influenced failures, such as a lack of standardization of materials across the hospital’s sectors and devices that fit in any input. Naturally, these do not exempt students from diverting their attention but constitute a barrier that, if eliminated, can prevent errors.

The reports show that the system’s factors are more responsible for triggering an incident than human factors. For example, miscommunication within the health team and a lack of protocols and standardization influenced the involvement of students in incidents. Additionally, the reports highlight that a lack of teamwork, overload of activities, not trusting the students, and personal factors favor students to get involved in risky situations, making them second victims.

The occurrence of incidents can be understood from two perspectives. The first focuses on an old and generalized thought, which identifies the individual responsible for errors based on unsafe actions, such as inattention or forgetfulness. From the second perspective, human beings are considered fallible, and incidents may happen regardless of the healthcare institution. Therefore, such events are seen as a consequence of systemic failures rather than originating from human factors^(14,15)^.

The author argues that although we cannot change the human condition, we may change the conditions in which human beings work. In this approach, barriers and defenses play a central role in error prevention. High-tech systems have many defensive layers, which must be used to make healthcare work environments safer^(16)^.

In the same way that health institutions should not skimp on alarms, physical barriers, human barriers, checklists, and automatic shutdowns to protect patients and professionals, educational institutions should invest in strategies to prevent students from becoming involved in patient safety events and encourage this topic to be taught early on^(16)^.

In addition to systemic failures, the students’ reports show that the teaching of the pillars of patient safety has weaknesses and is influenced by a punitive culture within the university context. As a result, they feel under pressure not to make mistakes, and this thought remains within them when they reach the program’s final stages, a time when they find themselves alone, without the supervision and monitoring of professors, and experience a paralyzing feeling that influence their resourcefulness within the internship^(17)^.

The fear of making mistakes and a perception that they cannot err show how this issue is still taboo in academic education and heavily influenced by strong negative safety or punitive culture^(18,19)^. The punitive culture is related to a tendency of managers and employees to judge and try to find the culprit of the events, thus, weakening the development of a patient safety culture and leading individuals to omit their errors and underreport. Hence, social desirability creates a false feeling in individuals that errors do not happen or should not happen in health institutions.

However, admitting that patient safety incidents happen is the first step to avoiding them. One in seven patients experiences safety incidents, impacting patients and families (first victim), healthcare providers (second victim), and healthcare services (third victim)^(8,20,21)^. However, the attitude professors adopt during activities in the hospital setting may be responsible for demotivating and traumatizing students, leading to poor communication and putting students at risk. Therefore, the trust established between professors and students is essential for clarifying doubts and avoiding errors in the care process. Additionally, the professors’ proper posture and support are essential to minimize distress and traumatic consequences arising from a patient safety event.

Most nursing students were insecure about the performance of their duties. From their perspective, this insecurity stems from the opportunity to perform procedures during internships, decreased internship workload, and a gap between the topics covered in theory and what is addressed during practice. These factors increase nursing student’s exposure to incidents when coupled with those in the hospital setting, such as workload and inadequate supervision^(22)^.

Addressing the possibility of errors, learning from them, and reporting them can support the students’ training process and prepare them to be professionals who understand and avoid the occurrence of errors and manage conflicts when involved in these events^(23)^.

Therefore, students should be encouraged through the communication of experiences with safety incidents. Additionally, a fair culture should be implemented in higher education institutions. Therefore, students should be adequately prepared for clinical settings and learn to talk openly about their mistakes, with the support of educators who should work on the causes of incidents and consider the system’s vulnerabilities rather than blame students^(16)^. Moreover, the reporting of patient safety incidents and supportive policies should be instituted by universities and transmitted to nursing students^(16,22)^.

Finally, this study’s limitations include the participants’ answers, which may have been influenced by bias, considering the topic involves feelings and experiences that have the potential to compromise the students, even though they were ensured their information would remain confidential. Additionally, because this is a cross-sectional study, memory may have caused forgetfulness or gaps in some responses. However, this study invites other researchers to analyze the effects of the second victim phenomenon among nursing students from a longitudinal perspective.

## CONCLUSION

This study revealed that nursing students might become second victims due to unsafe healthcare systems. The students identified that poorly planned systems, lack of standardization and established flows across all hospital units, lack of trust within the staff and poor communication, are factors exposing them to risk.

Additionally, the students feel insecure because the clinical setting is a complex and dynamic environment. The teaching-learning process was also reported as contributing to their insecurity. From their perspective, not all the content required in a clinical context is taught in the classroom. Hence, students are more likely to become second victims when these environmental factors are coupled with a lack of open communication with professors caused by a strong punitive culture.

Addressing patient safety in the nursing program can support the development of a patient safety culture by encouraging reporting, admitting the possibility of errors, and learning from them, all strategies that can mitigate the effects of the second victim phenomenon.
